# Cultural Welfare as a relational form of urban social innovation: evidence from the city of Bologna

**DOI:** 10.3389/fsoc.2026.1934171

**Published:** 2026-08-31

**Authors:** Roberta Paltrinieri, Orkide Izci

**Affiliations:** Department of the Arts (DAR), Alma Mater Studiorum – University of Bologna, Bologna, Italy

**Keywords:** Bologna, cultural welfare, medium-sized cities, proximity, relationality, social cohesion, third sector, urban social innovation

## Abstract

Medium-sized European cities, often celebrated for their quality of life and civic culture, are increasingly experiencing subtle socio-economic and spatial pressures, such as demographic aging, housing strain, and commodification of urban centrality, that erode everyday proximity and social cohesion without surfacing as visible emergencies. Drawing on 26 in-depth interviews with third-sector and cultural organizations across Bologna, this article argues that Cultural Welfare represents a relational form of urban social innovation. Situated within the second-welfare framework, Cultural Welfare emerges as a relational infrastructure in which artistic practices generate shared meanings, recognition, encounters, and the capacity to aspire to alternative urban futures, producing proximity, relations of trust and care. The analysis identifies two interconnected mechanisms through which these initiatives contribute to addressing socio-spatial inequalities and promoting inclusion by widening access to symbolic and relational resources, and to culture-led urban regeneration that reframes safety and liveability as emergent properties of cultural presence rather than solutions supplied through control, surveillance, or market-led redevelopment. These effects, as reported by interviewed organizations, include reclaimed spaces, a greater sense of safety and belonging, and denser networks of mutual care and solidarity, operating at neighborhood and city scales to complement rather than substitute for structural policies. Reading these practices through relational approaches to urban social innovation, the article contends that the specific orientation of the social enacted by Cultural Welfare is the regeneration of the relational fabric eroded by contemporary individualization, extending scholarship on socio-ecological transitions toward a cultural and relational dimension that dominant strands have left under-theorized.

## 1 Introduction

Urban areas play a central role in contemporary socio-ecological transitions, concentrating social fragility and functioning as key sites of social inequality and exclusion as well as potential ecosystems for social innovation. As highlighted by the World Cities Report 2022, urban poverty and inequality remain among the most intractable and complex problems confronting cities today (UN-Habitat, 2022). While much urban scholarship has focused on large metropolitan contexts, comparatively less attention has been paid to small and medium-sized cities (Servillo et al., 2017), despite their specific institutional configurations and their capacity to function as ecosystems for relational, place-based and civic forms of social innovation emerging through everyday cultural and social practices (Moulaert et al., 2013).

Across Europe, medium-sized cities that are often celebrated for their quality of life, civic participation and cultural vibrancy are increasingly experiencing socio-spatial pressures. Rising housing costs, demographic aging, precarious work trajectories and the growing commodification of urban centrality are reshaping everyday life in ways that are not always immediately visible. More often, they surface as subtle yet persistent tensions: older residents living in isolation, children navigating unequal educational environments, migrant marginalization, and contested uses of public space.

Bologna is frequently portrayed as a model of civic engagement and progressive local governance. The city has a long-standing tradition of cultural production, dense associative networks, social movements and participatory policy-making, and is administratively divided into six districts (quartieri). In recent years, the municipality has further institutionalized forms of shared administration, encouraging co-programming and co-design between public authorities and third-sector actors. Yet, as in many European cities, the social fabric of Bologna's districts reflects tensions connected to broader socioeconomic transformations. Pressures linked to tourism, commercial reconfiguration and uneven access to housing intersect with demographic change and economic precarity, generating differentiated experiences of vulnerability across age groups, gender and people with migration background.

Against this background, the article addresses one principal research question: how, and under what conditions, do cultural and artistic practices promoted by third-sector organizations operate as relational forms of urban social innovation in a medium-sized European city such as Bologna? This question is developed through three subordinate lines of inquiry: first, through which mechanisms cultural and artistic practices reduce inequalities and widen access to symbolic, relational and material resources; second, how such practices contribute to culture-led regeneration of public space and to reframing urban safety and liveability; and third, under what conditions this transformative potential varies across Bologna's heterogeneous urban fabric, and what limits and contradictions it encounters.

Drawing on the capability approach (Sen, 1999; Nussbaum, 2011), which fosters relational interdependence, social cohesion and inclusion, Cultural Welfare operates through the reweaving of social ties, the activation of community networks and the co-production of solutions to emerging needs. It is grounded in participatory and proximity-based practices that encourage citizens not merely to receive services, but to act as contributors to shared processes of community development and to the collective imagination of an alternative future. In this sense, Cultural Welfare functions as an enabling infrastructure that expands opportunities for participation, recognition and agency, rather than as a compensatory tool intervening on specific categories of fragility. Following Reckwitz (2017, 2020), we treat the creativity mobilized by these practices as ambivalent rather than straightforwardly emancipatory: alongside its capacity to build shared meaning, it also operates as a generalized expectation of originality and distinctiveness that can shade into selective valorization. This micro-sociological ambivalence has a macro-structural counterpart in the political economy of the “creative class” and culture-led regeneration (Florida, 2002; Peck, 2005), where the same demand for distinctiveness is absorbed into urban growth strategies that can reproduce, rather than mitigate, socio-spatial inequality.

Empirically, the argument is grounded in 26 in-depth interviews with third-sector and cultural organizations mapping Bologna's Cultural Welfare ecosystem (Section 3), read through a relational approach to urban social innovation (Moulaert et al., 2013; McFarlane et al., 2023).

In this article, we argue that, within this evolving context, Cultural Welfare emerges not as a sectoral intervention but as a reconfiguration of welfare itself. Rooted in the broader paradigm of “second welfare” (Maino, 2023; p.55) and community welfare, Cultural Welfare moves beyond a reparative or assistance-based logic. It repositions culture as a structural dimension of collective wellbeing and agency (Manzoli and Paltrinieri, 2021). Rather than treating cultural activities as complementary to social services, this approach frames culture as a generative resource that can strengthen social cohesion, enhance individual and collective capabilities, and foster active citizenship.

In this context, cultural initiatives do not position themselves primarily as corrective responses to fragility. Rather, they act as generative processes in which individual and collective capacities can be imagined and cultivated. Through everyday practices ranging from shared cultural initiatives and artistic production to informal encounters in public and semi-public spaces, these actors foster forms of relationality and proximity that enable participants to develop voice, confidence, reciprocal trust and a sense of belonging to the community, the district and the city. Vulnerability, in this sense, is not treated as a fixed attribute of predefined groups, but as a relational and universal condition, one that concerns everyone to some degree, yet is unevenly intensified and distributed through institutions, economic circumstances, social relations and regimes of recognition; participation and cultural experience can transform how, and how unequally, this condition is lived, without eliminating it. Rather than replacing the traditional welfare system, these initiatives operate within a broader ecosystem of community-based Cultural Welfare. Their contribution lies in strengthening the connective fabric of the territory: facilitating encounters across generational, gendered and migratory differences; widening access to (central) urban spaces; and expanding opportunities for active involvement in collective life. In doing so, it is not only their continuous on-site presence that reshapes the meaning of urban space and, by extension, of the city itself; it is the social and intrinsic value of cultural and artistic activities that makes this reconfiguration possible. Through the symbolic, expressive and relational qualities specific to cultural practice, these actors redefine the city not as a site of consumption, but as a lived environment where these very practices sustain relational proximity, countering the growing individualization and social isolation that increasingly characterize contemporary urban life.

By foregrounding the everyday work of cultural and third-sector actors, this article contributes to current debates on the socio-ecological transition of urban areas introduced above, and specifically to how that transition unfolds in medium-sized European cities. Rather than approaching transition exclusively through environmental or infrastructural lenses, it focuses on how the cultural and artistic practices enacted by these actors reshape the city, expanding access to cultural participation and advancing a more democratic cultural citizenship. In contexts marked by demographic shifts, housing pressures, commercial transformation and uneven access to symbolic and material resources, urban transition unfolds not only through planning strategies but also through the reconfiguration of everyday forms of coexistence and access to what a city offers. From this perspective, the socio-ecological character of urban transition can be understood as the interdependence between social cohesion and urban sustainability: the capacity of a city to remain liveable over time depends not only on environmental policies, but also on the strength of its relational infrastructures and on the distribution of opportunities for participation, recognition and inclusion. Cultural Welfare thus becomes a lens through which to observe how urban belonging, centrality and collective capacities are renegotiated in times of socio-spatial transformation.

## 2 Theoretical framework: Cultural Welfare as a relational form of urban social innovation

Throughout this article we use a cluster of related but distinct terms, cohesion, relationality, proximity, trust and care, that recur across the Cultural Welfare literature without always being clearly distinguished. Following Prandini and Villani (2025), we treat social cohesion as combining an ideational component, the sense of belonging and shared meaning that cultural participation generates, and a relational component, the observable ties and patterns of interaction among individuals, groups and institutions that such participation sustains. Relationality, proximity, trust and care refer, in our usage, to specific facets of this relational component: proximity to the physical and social conditions of co-presence, trust and care to the qualities of the ties that recurring cultural practice helps to produce, and cohesion to their aggregate outcome at the level of a neighborhood or city. On this basis, we argue that Cultural Welfare can be productively understood as a relational form of urban social innovation: one that responds to the individualization and social isolation characteristic of contemporary urban life (Paltrinieri, 2022) by mobilizing culture as an infrastructure for relationality and creating proximity and social cohesion, in the specific senses just outlined. This section builds that argument in three steps. It first situates Cultural Welfare within the second-welfare and capability paradigms; it then sets out the conceptual architecture of urban social innovation and its constitutive urban mechanism; finally, it makes explicit the nexus through which Cultural Welfare can be read as a culturally mediated instance of that mechanism.

### 2.1 Cultural Welfare: from a health-oriented to a relational and collective paradigm

Under the broader umbrella of Cultural Welfare are good practices that promote empowerment and subjective wellbeing, individual social capital relating to relational aspects, the combating of inequalities in health and access to resources, the accompaniment of active aging, and the prevention of psychophysical decline. While earlier, health-oriented understandings framed Cultural Welfare primarily as a means of increasing cultural participation in order to improve the quality of life of physically and psychologically fragile individuals, this understanding has since shifted (Allegrini et al., 2025). We owe this shift to the medical sociologist Aaron Antonovsky, who already in 1979 argued that, in terms of prevention, it is more important to focus on resources and people's ability to create wellbeing than on the classical focus on risks and illnesses. It is precisely this turn, from deficit and pathology to resources, capabilities and the active creation of wellbeing, that grounds our own approach.

In this article, we situate Cultural Welfare within the broader framework of “second welfare” (Maino, 2023, p. 55). While traditional welfare is primarily organized around universal public services and public funding, second welfare refers to a complementary set of interventions that are not exclusively financed or managed by the public sector, but are co-produced by local authorities, third-sector organizations, foundations and private actors. These arrangements are explicitly oriented toward social innovation, prevention and the strengthening of people's capabilities, rather than the mere compensation of risks. Cultural Welfare represents a specific expression of this second welfare; it mobilizes cultural institutions, community organizations and artistic practices to generate wellbeing, inclusion and empowerment through locally rooted, multi-actor collaborations.

According to the Cultural Welfare perspective, caring for the individual depends on a systematic collaborative relationship among professionals from different disciplines and, above all, on the integration of purposes among the institutional systems of health, social policy, and the arts and culture (Fulco, 2022). Cultural and artistic experiences such as dance, theater, painting, and visits to museums or cultural heritage sites are in themselves resources for wellbeing (Bungay and Clift, 2010) generated through participation within communities rather than delivered as a service. From this perspective, a more collective and community-oriented understanding of wellbeing concerns the innovative form of welfare and the broader processes of social and cultural innovation (Andersen and Pors, 2016). Importantly, the relationship between social innovation and cultural processes follows a double track. On the one hand, cultural values and processes are the fuel for social innovation; on the other hand, social innovation can be defined as the ability to achieve certain common goals through creative means (Manzini, 2018).

At the center of Cultural Welfare, from a more collective perspective, lies the theme of agency and the cultural capacitation of communities, that is, the capacity to generate alternative landscapes, following Appadurai's (1996) theory of imagination, to promote culturally oriented and therefore transformative social action. This vision implies a way of thinking and acting, individually and collectively, that is creative, collaborative, responsible and capable of virtuously impacting upon the forms of living and organizing, in the perspective of the communities of practice proposed by Wenger (1998).

As a consequence, Cultural Welfare must be reinterpreted by synergistically promoting cultural practices and their networking. This means promoting the co-planning of circular subsidiarity, at the center of which there must be the production of cultural capacities. In this way, redistribution processes aim to increase awareness of which model of society one wants to belong to. If one accepts the assumptions of this different approach to culture, what Cultural Welfare must aim at is a process of growth of cultural participation, where the ultimate goal of its activation is the promotion of cultural citizenship (Klaus and Lünenborg, 2012). Here we do not mean the legal dimension of citizenship, but cultural citizenship as access to knowledge, communication and, above all, to the social responsibility that derives from it, with a view to the construction of imaginaries and communities. Specifically, we propose a model of Cultural Welfare whose salient feature is that it is neither reparative nor standardized, but rather a type of welfare capable of producing flexible, customized and multidimensional responses, aimed at promoting cultural capital while at the same time imagining alternative futures and possible scenarios.

### 2.2 Urban social innovation: definitional structure

Situating Cultural Welfare in relation to urban social innovation requires engaging with the conceptual structure of the latter. Although there is no single, agreed definition of social innovation (Mulgan et al., 2007; Pol and Ville, 2009; Rueede and Lurtz, 2012; Moulaert et al., 2013), systematic reviews converge on three core components observable across its many formulations: it is a process that concerns new social relations in systems or structures; it produces an outcome of new social value by meeting needs or addressing socially relevant problems; and it operates across multiple spatial scales: micro, meso and macro (Ardill and de Oliveira, 2018). The same scholarship distinguishes a polarity between a radical interpretation, which targets the transformation of power relations, socio-spatial justice and the empowerment of marginalized groups (Moulaert et al., 2005), and a complementary interpretation, more congruent with existing political and market arrangements, which seeks to meet societal needs more efficiently and risks sliding into a “caring neoliberalism” that leaves dominant structures unchallenged (Mulgan et al., 2007; Ardill and de Oliveira, 2018).

In its most influential formulation, social innovation is held to possess transformative potential to create more inclusive places grounded in respect, empathy, openness and tolerance (Moulaert and MacCallum, 2019), through meeting needs; generating new forms of ecological, social or institutional relations; and collectively empowering people, particularly the marginalized (Moulaert et al., 2013). From this perspective, the urban dimension functions as a mechanism for bringing different people into proximity, such that the central challenge of innovation becomes that of translating proximity into normative relations of respect and care (McFarlane et al., 2023). It is precisely this passage from spatial co-presence to relational care, we argue, that Cultural Welfare enacts by cultural means: culture, by virtue of its intrinsic social value as a generator of shared meanings, becomes the relational infrastructure through which physical proximity is woven into trust, recognition and care.

Recent work, however, warns against treating urban social innovation as a fixed set of normative principles. McFarlane et al. (2023, p. 338) argue that the “urban,” the “social” and “innovation” are not pre-given categories but are relationally co-constituted by differently positioned actors, who variously claim, pursue or resist them. From this perspective, the analytical task is not to measure initiatives against a predefined ideal of “progressive” innovation, but to “drill down” into how, in a given context, actors bring these terms into relation (McFarlane et al., 2023, p. 346). This relational and situated reading is significant for our case for two reasons. First, it foregrounds that the transformative potential of urban social innovation often lies less in the production of novel outputs than in the slow, collective and frequently unrecognized work of sustaining relations, conditions and communities over time. This resonates with recent work on grassroots initiatives in Bologna, where actors reinterpret circular and regenerative practices beyond their technical or economic function, reframing them as processes rooted in care, solidarity and territorial wellbeing (Landi et al., 2026); here too, community relations and relational proximity are themselves the value being generated and regenerated, rather than a by-product of innovation. Second, this reading allows us to position Cultural Welfare as a distinctive, culturally mediated configuration of the urban–social–innovation relation, whose specific orientation of the social we make explicit below. What specifically qualifies this configuration as *cultural* welfare is that the relational fabric it mobilizes is not a pre-given stock of social capital, but is itself generated through the symbolic and expressive force of artistic and cultural practices. It is the capacity of art and culture to create shared meaning, enable expression and open spaces of encounter that activates proximity, trust and relational density in the first place. The relational dynamics on which social innovation depends are, in this sense, co-produced through cultural mediation rather than simply presupposed by it. Read in this way, this relational reading also resonates with research suggesting that urban social innovation tends to emerge less from top-down institutional mandates than from situations of uncertainty in which dense networks of social capital and trust, here culturally activated, allow actors to respond flexibly to changing local conditions (von Schnurbein et al., 2023). Its transformative power, in this view, is enabled by the values of solidarity, reciprocity and association, and rests on collaborative processes that cross organizational and sectoral boundaries (Ziegler, 2017; Moulaert and MacCallum, 2019).

### 2.3 Cultural Welfare as practice and relational infrastructure

Read together, these two bodies of literature converge on a single insight that frames our analysis. If urban social innovation, in its transformative register, works by regenerating the relational infrastructure that contemporary urban processes erode, then Cultural Welfare is a specific mechanism through which this regeneration occurs since it mobilizes cultural participation and capacitation to convert urban proximity into relations of trust, care and cultural citizenship. In this sense, Cultural Welfare does not merely resemble urban social innovation; it enacts its constitutive relational logic by cultural means, offering a situated response to the crisis of individualization that pervades contemporary life (Paltrinieri, 2022). It is this relational mechanism, culture as an infrastructure for relationality, that we trace empirically in our case study. In doing so, the article also positions Cultural Welfare as an extension of urban social innovation scholarship toward a cultural and relational dimension that the dominant, neoliberal and service-oriented strands of the field have left comparatively under-theorized.

Yet this relational logic of urban social innovation is not enacted in the abstract: it is carried by concrete cultural and artistic practices, and what we mean by “practice” needs to be made explicit before we can trace it empirically. Following Reckwitz's (2002, p. 249) theory of social practices, we read cultural participation through two registers at once. Third-sector organizations supply the intentional purpose, resources and institutional continuity, an organizational program, without which sustained cultural action would not take place at all; but whether that action actually produces relational infrastructure is a further, practical question. Reckwitz defines a practice as “a routinized type of behavior which consists of several elements, interconnected to one another: forms of bodily activities, forms of mental activities, “things” and their use, a background knowledge in the form of understanding, know-how, states of emotion and motivational knowledge.” Read this way, relationality is not an attribute of an organizational program as such, but an empirical outcome of how these elements are recurrently and materially held together within it: the material organization of spaces and objects (a wall, a square, an artistic technique), the embodied and relational know-how of facilitation and mediation, the emotional investments and shared understandings that make certain forms of access and membership meaningful. Crucially, it is the routinized character of this configuration, its recurrence over time, rather than the organizational program's stated intention alone, that distinguishes a practice from an occasional event. Relationality is therefore not read off from the mere existence of a cultural initiative or its declared aims; it is reconstructed from evidence of recurring, materially organized encounters: the same wall repainted and maintained, the same square animated week after week, the same facilitators returning. This distinction is not only analytical: interviewees themselves draw a similar line when they describe their cultural and artistic activity as oscillating between an emergency-driven, *ad hoc* register and a more structural, systemic one.

This relational reading, however, should not be taken as inherently emancipatory. Following Reckwitz (2017, 2020), we treat creativity itself as ambivalent: alongside its capacity to generate shared meanings and recognition, it also operates in late modernity as a generalized social expectation, a dispositif that requires individuals, organizations and cities to appear original, authentic and distinctive, and that can shade into the selective valorization and aestheticization of both people and places.

## 3 Materials and methods

This study adopts a qualitative research design developed through collaborative, stakeholder-informed sampling, within a broader partnership between the municipal administration, the Scuola Achille Ardigò and the University of Bologna, grounded in principles of shared governance that characterize Bologna's community welfare model (Manzoli and Paltrinieri, 2021). Local actors, principally district officials and third-sector organizations, were involved in co-constructing the sample and the interview instrument, while the research questions, the coding and interpretation of the material, and the conclusions presented in this article were developed by the authors. Community actors were not directly involved in validating thematic categories or discussing contradictory findings during the analytic phase. Findings were nonetheless returned to the community: a report was distributed by the Quartiere and the Scuola Achille Ardigò, and results were presented at a public event organized by the Scuola Achille Ardigò, attended by residents and participating organizations, where the feedback received confirmed the critical issues presented and will inform our future research. This approach allowed the study to remain closely attuned to the city's social dynamics while maintaining methodological coherence and analytical independence.

The focus on the city of Bologna was motivated by its character as a medium-sized European city that concentrates multiple and coexisting forms of socio-spatial transformation. Although widely recognized for its tradition of social movements and civic culture, the city presents both visible and less visible forms of fragility, including tensions in public space, aging residents, working poor migrants, educational inequalities and changing patterns of touristic, commercial and residential dynamics that are unevenly distributed across its different urban areas.

The qualitative sample of 26 organizations was not drawn from a pre-existing list handed to us by the municipal administration, but co-constructed together with Bologna's district offices, in particular the Ufficio Reti (*Networking Office*) with the research instrument itself co-designed through this same process. Initial mapping proceeded area by area for each district, together with officials from *Ufficio Reti* of different districts, prioritizing areas identified as particularly fragile in terms of both social need and the scarcity of institutional presences. Beyond this institutionally co-constructed core, the sample was extended through snowball referrals: interviewees themselves indicated further organizations and informal groups active in the territory, including some not already recognized within the municipality's own network of contacts, allowing the study to reach beyond organizations already visible to public institutions. However, we recognize that the strong institutional embeddedness of the research creates a real risk of selection bias toward organizations already recognized by public institutions; the snowball component of the sampling strategy was designed specifically to mitigate this risk, though it cannot eliminate it entirely, and more conflictual, informal or short-lived initiatives operating outside any institutional network may still be under-represented in our data. Across Bologna's six districts, the resulting sample privileges depth over uniform territorial coverage, concentrating on a smaller number of areas rather than distributing interviews evenly; the sample is therefore purposive rather than representative in a statistical sense, oriented toward theoretical saturation within each area studied rather than exhaustive citywide coverage. Given this sampling strategy, we use our data to reconstruct a Cultural Welfare ecosystem within the specific areas studied, rather than claiming exhaustive coverage of Bologna as a whole.

A qualitative sample of 26 organizations was selected to map Bologna's Cultural Welfare ecosystem (Table 1). The selection included associations, residents' committees, cooperatives, neighborhood houses (Case di Quartiere, publicly owned spaces managed by third-sector associations under agreement with the district administration) and a parish-based youth space, actively engaged in cultural production, community-based initiatives, artistic practices and neighborhood-level programming across different urban areas of the city. Together, these organizations represent a heterogeneous ecosystem of actors working with different social groups, including older residents, migrant communities, children and families.

**Table 1:** Interviewed organizations (*N* = 26): anonymized description and domain of activity.

ID	General description	Practices/activities (not exhaustive)	Main sector/domain
**INT01**	Voluntary association rescuing books destined for pulping and recirculating them free of charge across the city.	Book recovery and free redistribution via book-stations in public and semi-public spaces; presence at city markets; a mobile book van reaching under-served areas.	Culture/reading/redistribution
**INT02**	Association caring for and reactivating a centrally located public green area affected by decay.	Cultural programming and stewardship of a shared green space; community reappropriation against drug-dealing and neglect.	Urban regeneration/public space
**INT03**	Residents' and shopkeepers' committee promoting an older central neighborhood and its public realm.	Community animation, social solidarity initiatives, care of common goods, neighborhood promotion.	Community/neighborhood committee
**INT04**	Cultural association promoting play and game culture, with social-support activities.	Game-design and board-game workshops, role-play sessions for all ages, itinerant game libraries, collaborations against social marginalization.	Play/culture/inclusion
**INT05**	Association promoting philosophical dialogue as an educational practice fostering critical thinking.	Dialogic philosophy workshops in schools, festivals, libraries and museums; civic and corporate training; annual philosophy festival.	Education/culture
**INT06**	Neighborhood social-recreational center, now a neighborhood house (Casa di Quartiere), oriented to psycho-physical wellbeing.	Reading and film groups, theater, choir, gymnastics, psychological support, digital-literacy courses for those at risk of digital divide, environmental education.	Community/wellbeing/aging
**INT07**	Neighborhood house (Casa di Quartiere) hosting a plurality of partner associations within one shared community hub.	Intergenerational and intercultural programming, social-portering services, music and creative workshops, management of shared spaces.	Community hub/multi-actor
**INT08**	Third-sector body managing several socio-educational and sports facilities across the district.	Socio-educational projects for young people, after-school and creative laboratories, sports facility and community spaces with an inclusive approach.	Socio-educational/sport/community
**INT09**	Residents' committee engaged in neighborhood promotion and proximity work.	Community-building, proximity initiatives, care of the local area and collective life.	Community/neighborhood committee
**INT10**	Residents' and traders' committee for the regeneration of a central area with strong potential.	Community animation, activation of residents and local businesses, promotion of a shared vision for the area.	Community/neighborhood committee
**INT11**	Association active in the field of disability, within a community-hub project, offering a listening and meeting space.	Listening point and meeting place with a café, cineforum, exhibitions, networking with other local associations.	Disability/inclusion/community
**INT12**	Feminist women's association engaged against exclusion, inequality and violence against women.	Political-cultural education, labor-market orientation, digital literacy, reading promotion, school workshops; co-management of a public garden.	Gender/empowerment/rights
**INT13**	Cultural association promoting healthy lifestyles and psycho-physical wellbeing.	Talks and seminars with health professionals, expert-led courses, cultural visits, outdoor physical activities, often with a local social-recreational center.	Health/wellbeing/prevention
**INT14**	Feminist association working on inclusion, anti-violence and the cultural heritage of women.	Documentation center, schools of politics, affectivity and sexuality education ateliers, cultural programming; co-management of a public garden.	Gender/culture/rights
**INT15**	Neighborhood association fostering human and solidarity ties through street and area regeneration.	Read-aloud events, theater, cinema, talks and a summer cultural season; collaboration agreements for the requalification of a street and a green area.	Urban regeneration/culture
**INT16**	Independent bookshop, artisanal printing house and small publisher.	Promotion of illustrated publishing and artisanal printing; atelier for residencies, workshops and educational activities for children, students and adults.	Culture/publishing/arts
**INT17**	Social-promotion association diffusing music and arts education with strong attention to inclusion.	Courses and workshops for all ages, music therapy, inclusive ensemble and choral practice, intergenerational projects.	Music/inclusion/wellbeing
**INT18**	Association working with contemporary action-theater for community wellbeing.	Theater promotion, training and improvisation; affiliation to local solidarity-theater networks; poetry and improvisation laboratories.	Theater/community/wellbeing
**INT19**	Public neighborhood library within the municipal libraries and cultural-welfare system.	Reading promotion, cultural programming, learning and meeting space for the local community.	Culture/reading/public service
**INT20**	Parish-based youth space functioning as an educational and aggregative setting open to all youth regardless of faith.	Continuous supervised presence, recreational and educational activities for minors and adolescents; occasionally hosting supervised meetings for vulnerable minors.	Education/youth/aggregation
**INT21**	Association engaged in educational activities and the contrast of educational poverty.	Educational and aggregative activities for children and families; community-based learning support.	Education/inclusion
**INT22**	Neighborhood house (Casa di Quartiere) offering proximity-based social and cultural programming.	Cultural, recreational and social activities; a community meeting place rooted in the neighborhood.	Community hub/proximity
**INT23**	Residents' committee engaged in proximity and the care of public space in a peripheral area.	Community-building, neighborhood animation, attention to liveability and shared use of public space.	Community/neighborhood committee
**INT24**	Association for the care and animation of an urban green area.	Stewardship of a park, environmental and cultural activities, community events fostering encounter.	Urban regeneration/public space
**INT25**	Work cooperative active in social innovation, inclusion and cultural production.	Cultural and inclusion projects, collaborative and community-oriented activities, social-innovation initiatives.	Social innovation/culture/inclusion
**INT26**	Grassroots sports club promoting sport for all and social inclusion.	Inclusive sport activities, youth involvement, community-based participation through sport.	Sport/inclusion/community

Between October 2024 and May 2026, 26 in-depth semi-structured interviews were conducted by the authors with representatives of the selected organizations, involving a total of 41 participants, as in some cases more than one representative from the same organization took part. Whenever possible, interviews took place on-site within the organizations' spaces, enabling direct observation of their environments and ongoing activities. In a limited number of cases, interviews were conducted online due to logistical constraints. Interviews lasted between 50 and 75 min and explored organizational histories, current projects, interpretations of culture in relation to wellbeing, participation in local networks, perceived challenges and the tools adopted to assess impact. The semi-structured format allowed participants to elaborate beyond the predefined questions, often leading to extended conversations and informal exchanges that enriched the empirical material. All interviews were audio-recorded with participants' consent and subsequently transcribed verbatim with the help of specific transcriptions software. In one case, the interviewee did not consent to audio recording; the corresponding material is therefore drawn from contemporaneous field notes and is reported in paraphrase rather than as verbatim quotation. In presenting the empirical excerpts throughout this article, all identifying information, such as the names of associations, projects, or specific organizational references, has been deliberately anonymized. This choice responds both to ethical considerations regarding the privacy of participants and to explicit requests made by several organizations and institutional actors to avoid public exposure or potential misinterpretation of their statements. Organizations are referred to through codes (INT01–INT26) and described in Table 1 only through the general nature of their activity without using any kind of visual territorial mapping since Bologna is a medium-sized city and it would not be difficult to guess the association indicated on a map. All excerpts originally in Italian were translated into English by the authors with the assistance of artificial intelligence tools.

### 3.1 Data analysis

Initial analytical categories were informed by the theoretical framework of Cultural Welfare organized around social, participational and relational dimensions, while additional themes emerged directly from the empirical material. The transcripts were analyzed through reflexive thematic analysis (Braun and Clarke, 2019, 2021). Authors coded the material manually, working first with descriptive codes that stayed close to participants' own categories. In a second stage, these codes were coded into broader analytic themes, recurring patterns and categories related to cultural welfare practices, meanings, and organizational dynamics including social cohesion, proximity and social relations.

Crucially, however, these categories were not treated as discrete functional domains but were read relationally: the analysis attended less to *what* Cultural Welfare delivers than to *how* the cultural and artistic practices reconfigure social relations and compose the urban as a lived, contested and relationally produced space. Among the multiple and interconnected domains through which Cultural Welfare manifests, this article concentrates on those that most clearly illuminate its urban character, re-reading them through the perspective of urban social innovation understood as a fundamentally relational form: one whose innovativeness lies not in cultural outputs as such, but in the way culture is brought into intersected relations with the spatial, social and institutional registers of city life. This relational orientation shaped the analysis at every level. Rather than coding cultural initiatives as bounded interventions, authors observed how they operate as mechanisms for reducing inequalities and promoting social inclusion, expanding access to symbolic, relational and material resources within the city, and how they contribute to the regeneration of urban spaces through collective reflexivity, participation and the production of inclusive public spaces that strengthen community ties. Urban transformation is therefore understood not primarily as physical redevelopment or market-driven renewal, but as a relational reconfiguration of social relations and forms of collective agency and care within the urban fabric. In this, we draw on relational approaches to urban social innovation (McFarlane et al., 2023), for which “urban,” “social” and “innovation” are not fixed analytical categories but are co-created through situated practices and actor configurations. Particular attention was therefore paid to how interviewees themselves produced the urban: to how they described socio-spatial fragilities, (contested) uses of public space, relations of proximity and participation, and their effects. The city is approached not as a geographical container but as a relational ecosystem in which cultural practices, community networks and institutional arrangements intersect and mutually shape one another.

Consistent with this relational framing, the analysis focuses on patterns of connection and recurring mechanisms rather than on individual organizations as discrete units. Rather than seeking statistical generalization, the study adopts a qualitative analytical approach identifying how Cultural Welfare practices operate as relational urban social innovation within broader processes of urban transformation, socio-spatial reconfiguration and inclusive participation.

## 4 Results

### 4.1 Reading Cultural Welfare as urban social innovation in Bologna

Cultural Welfare can be interpreted not only as a policy field linking culture and wellbeing, but as a relational infrastructure that promotes empowerment, wellbeing and the strengthening of social capital through cultural participation (Paltrinieri, 2025). It seeks to enhance people's wellbeing and their capacity to co-produce meaning through artistic and cultural practices. Situated within the broader framework of second welfare (Maino, 2023), it operates through multi-actor collaborations by mobilizing cultural institutions, third-sector organizations and civic actors in co-produced processes (Fulco, 2022) aimed at inclusion, capability-building and social cohesion. In this sense, Cultural Welfare can be understood as a form of social innovation insofar as it reorganizes relationships, activates collective reflexivity and addresses societal challenges through collaborative and participatory practices. Social innovation, as discussed above, is articulated along three interrelated dimensions that resonate closely with Cultural Welfare practices (Moulaert et al., 2005).

While these three dimensions specify what social innovation does, they say relatively little about where the “urban” comes in, and even less about the specifically cultural register through which it is enacted. Our contribution is to foreground the medium through which urban, social and innovation are brought into relation takes place: in the case of Cultural Welfare, it is artistic and cultural practices that compose the urban, the social and the innovative into a single configuration. This has a direct methodological implication for how we read Bologna. Rather than treating the city as a generic container, or assuming that “the urban” corresponds to one bounded neighborhood more authentically than another, we follow how cultural organizations themselves variously enact the urban through their artistic and cultural practices: for some, it is the immediate street or square they inhabit daily; for others, it is a looser, city-wide field of relations, networks and circulating practices that exceeds any single administrative boundary. Cultural Welfare, in this sense, does not presuppose a single spatial scale at which urban social innovation properly occurs; instead, its urban character emerges from the situated ways in which cultural and artistic practices allow organizations, residents and institutions to compose relations of proximity and care across Bologna's heterogeneous districts. Within this framework, Cultural Welfare can be read as a social innovation because it activates what we have defined as processes of cultural capacitation and cultural capital (Paltrinieri, 2025). It fosters shared practices that enable communities to interpret their present, re-signify their spaces and strengthen relational infrastructures. Its innovative dimension lies in the way it connects associations, residents, professionals and local institutions around cultural initiatives that generate symbolic, relational and social value simultaneously. Its strength lies not in “filling institutional gaps,” but in mobilizing cultural resources to reinforce social bonds, participation and shared responsibility across heterogeneous populations. This universal orientation is crucial: cultural welfare does not target only vulnerable groups, nor does it stigmatize fragility, but creates spaces in which different socioeconomic and generational groups can interact on an equal symbolic plane. This dimension becomes particularly significant in Bologna, a city marked by pronounced socio-spatial heterogeneity, whose fabric includes both areas of high socioeconomic capital and zones of greater fragility and social turnover, unevenly distributed across its six administrative districts.

While social innovation is not inherently tied to a specific territorial setting, its manifestation is always context-dependent (McFarlane et al., 2023). In Bologna, characterized by demographic change, commercial transformation, tourism pressures and evolving forms of urban coexistence that vary considerably from one district to another, Cultural Welfare takes shape as a relational infrastructure that reinforces proximity, recognition and everyday cooperation, even as the concrete configuration of “proximity” differs across the city. Organizations rarely describe their work as the execution of a pre-defined plan, but rather as an ongoing, improvised negotiation with the specific socio-spatial conditions of the area in which they operate. The following sections analyze how Cultural Welfare unfolds along two interconnected dimensions across the city: reducing inequalities and promoting social inclusion within a heterogeneous urban fabric, and fostering culture-led regeneration processes that strengthen social cohesion and reshape the lived experience of urban space.

### 4.2 Cultural Welfare to reduce inequalities and promote social inclusion

This domain highlights how Cultural Welfare initiatives across Bologna address inequalities experienced both socially and spatially. Organizations working with women, children, migrants, former detainees, elderly people and individuals facing different forms of vulnerability describe culture less as a compensatory service and more as an enabling resource and capability. In their accounts, cultural practices create conditions for participation in which people can access shared spaces without having to conform to pre-defined norms of competence, legitimacy or cultural capital. In this sense, Cultural Welfare becomes a mechanism for widening access to urban centrality, materially, through free or low-threshold activities, and symbolically, through recognition and the right to participate, to speak, to contribute and to be visible.

Reducible to material deprivation alone but concerning symbolic recognition, relational isolation, gendered discrimination, unequal access to cultural participation and intergenerational fragmentation. Second, they reorganize social relations by creating bottom-linked arenas where associations, schools, institutions and informal groups collaborate through shared cultural and artistic practices. Third, they generate empowerment by enabling participants to reinterpret their social position, acquire reflexive and expressive competencies and gain visibility within urban public space. This transformative dimension can also be interpreted through the lenses of recognition, capabilities and agency. As Fraser (2000) argues, social justice requires not only the redistribution of resources but also the recognition of marginalized identities and experiences. Many of the initiatives described below operate precisely at this symbolic level: they create arenas where women's histories, migrants' narratives or the voices of socially isolated individuals become publicly visible. Inclusion, in this sense, is not only about access, but about being acknowledged as a subject entitled to participate in the shared construction of meaning. At the same time, these practices resonate with Sen's (1999) capability approach, insofar as they expand individuals' freedoms to act, to express themselves and to engage in collective life. Cultural participation becomes a resource that enhances people's capability to imagine alternative trajectories and to inhabit urban space with greater confidence and agency. By widening both recognition and capabilities, Cultural Welfare, in organizations' own accounts, contributes to reducing inequalities not only materially, but relationally and symbolically.

One of the most significant areas in which this transformative potential emerges concerns gender equality and processes of women's emancipation. A women's association working on violence and empowerment described its approach as follows:

*We begin by bringing forward our experience as women. We present ourselves as an association whose history is one of resistance and which, above all, follows a path of emancipation, because our aim is to make women aware that they have a role in society, a role that must be built consciously, a role of work, of economic independence, of rights, and of participation in all the rights and future opportunities that a first-class citizen can have. This is very important, and we do it in different ways: through film screenings and sharing moments together like we are doing now, through theater, through writing, tools that help us deal with very personal and often painful issues, and to understand how women have come to be marginalized in society*. (INT12; translated from Italian by the authors with the assistance of artificial intelligence)

Emancipation is articulated in explicitly socioeconomic and civic terms, through work, economic independence, rights and full participation in public life and is pursued through concrete forms of relational activation and networks within the city. Beyond cultural awareness-raising, the association engages employers, human resource managers, anti-violence centers and local institutions in order to facilitate sustainable pathways of labor insertion for women exiting violence. This involves not only creating job opportunities, but fostering institutional awareness of the structural and everyday obstacles these women face, such as care responsibilities, lack of economic autonomy, mobility constraints and the absence of support networks. Cultural participation and collective reflection thus function as enabling infrastructures that generate both consciousness and practical access to resources. Through self-help groups and networks of mutual support, women are not only encouraged to confront the personal and psychological dimensions of violence, but are embedded in protective relational environments that enhance their capacity to navigate work, rights and public life.

Similarly, theater-based and participatory methodologies are used to surface implicit biases and foster reflexive change. One association working with contemporary action-theater (INT18) described a multi-year project on equal opportunities that addresses gender stereotypes through theater laboratories, creative facilitation, live role-playing and participatory arts, and that circulates these practices across universities, academies and companies. What is particularly significant is not only the methodological hybridity but the circulation of these practices across multiple institutional settings. Cultural Welfare thus becomes a multi-contextual relational infrastructure, aligning artistic intervention with educational and civic frameworks and enabling sustained collaboration across sectors.

In addition to structured interventions, informal cultural spaces also function as hubs of inclusion. A board-game association described how play operates as a relational catalyst:

*Playing helps you to step out of your comfort zone and the stress of the day. Many games are collaborative and help create stronger bonds. We have created an environment where beginners play with experienced players and people of different ages. We have provided a place to socialize for people who are very alone, often with difficult life stories. They sit with people they do not know, and create relationships*. (INT04; translated from Italian by the authors with the assistance of artificial intelligence)

In this context, play becomes a medium for trust-building and reciprocity. What may appear as leisure reveals itself as a subtle mechanism of social inclusion, enabling individuals experiencing isolation to access shared spaces without stigma.

The reduction of inequalities also emerges clearly in initiatives aimed at redistributing cultural resources beyond socially advantaged groups. An association engaged in free book distribution observed:

*When we go to peripheral areas of the city, the users are more fragile. People are almost surprised to find a book stand. There, the book is valued more. Some wrote “thank you for the books, it is not easy to find them for free.”* (INT01; translated from Italian by the authors with the assistance of artificial intelligence)

This testimony highlights how access to cultural goods remains socially stratified and how, in the account of this organization, Cultural Welfare can function as a form of symbolic and material redistribution, mitigating disparities in cultural capital. Finally, several organizations explicitly address disability, intergenerational isolation and adolescent withdrawal through inclusive artistic practice. A music association explained:

*We combined our cultural work with a stronger social vocation, developing music therapy and inclusion projects for fragile children and adults. In the orchestra, everyone is equal and everyone is essential. We also created laboratories for grandparents and children, where the elderly regain vitality and children learn self-control. For fragile adolescents, we provide non-judgmental spaces where they can express themselves freely*. (INT17; translated from Italian by the authors with the assistance of artificial intelligence)

Here, artistic practice suspends hierarchical distinctions and creates environments where participants are recognized as equally necessary to the collectivity. Inclusion is enacted not through targeted assistance alone but through shared participation in cultural production. Taken together, both structured interventions and informal practices, as organizations describe them, reveal how Cultural Welfare generates relational infrastructures that reduce inequalities by widening access to recognition, strengthening capabilities and expanding participation in urban public life. In dense central districts marked by socio-spatial polarization, these practices acquire particular relevance: they do not eliminate structural inequalities, but they reconfigure the conditions under which individuals and groups can inhabit urban space with dignity, voice and agency. Following Barca and Zabatino (2025), these episodes can also be read as instances of a “squarcio,” a rupture in prevailing common sense that unsettles taken-for-granted hierarchies of value and opens space for alternative ways of seeing and recognizing people. The same authors caution, however, that such ruptures are typically momentary and unstructured unless sustained by recurring, organized engagement reaching beyond a narrow circle of participants; read through the practice-theoretical lens introduced earlier, this is another way of stating that a rupture in common sense becomes a durable relational infrastructure only once it is routinized. These relational dynamics, however, do not remain confined to interpersonal interaction. As the next section illustrates, they also extend into the spatial and material fabric of Bologna's urban areas, where Cultural Welfare contributes to processes of urban regeneration.

### 4.3 Culture-led urban regeneration for social cohesion

A second set of findings concerns Cultural Welfare as a driver of culture-led urban regeneration oriented to social cohesion. In the interview narratives, ‘regeneration' is not primarily defined as physical redevelopment, nor as an aesthetic upgrading of the neighborhood. It is described as an everyday process of sustaining public space through artistic and cultural presence, care and the cultivation of shared routines. Several actors emphasize that urban liveability is shaped by whether streets, courtyards, parks and semi-public spaces are inhabited through cultural and artistic practices: programming events, animating a small square, bringing a park to life, or reclaiming neglected corners by cleaning walls and painting over grey, degraded surfaces. A recurring example is the collective creation of murals on bare or deteriorated walls, often realized together with children from local schools, residents and artists, who design and paint the images collaboratively. Crucially, these gestures are not experienced as maintenance or d9cor, but as expressive and symbolic acts through which residents and organizations produce shared meanings and inscribe them into the very fabric of the place. A wall painted by schoolchildren is no longer simply a surface to be kept clean: it becomes a shared narrative, a visible trace of collective authorship that ties younger generations to the space and transforms how the whole community perceives and inhabits it. It is through this cultural and artistic labor that value is restored to shared spaces and that they are reshaped into lively and welcoming environments for the whole community. In this sense, the neighborhood and, by extension, the city are not merely repaired but re-signified: the creation of common areas designed for encounter and the strengthening of belonging are pursued through the active, creative involvement of residents in the shared life of common goods (Allegrini et al., 2026). These practices reflect the social value of proximity: the wellbeing generated when people share space, interact and inhabit places together, which may also transform into spaces of resistance (Boschma, 2005; Pink, 2012; Manzini, 2021). In a context marked by growing individualization (Bauman, 2000; Beck and Beck-Gernsheim, 2002) and the “pluralization of life-worlds” (Berger et al., 1974; pp. 64–65), Cultural Welfare initiatives foster forms of physical closeness that have become increasingly rare. Culturally animated public spaces can foster informal social order and mutual trust, as people gather, interact and take shared responsibility for the places they inhabit. Organizations describe these dynamics as making public spaces feel safer, more inclusive and less vulnerable to deterioration (Dovey, 2016; Low, 2023), a perceived rather than independently measured effect. Proximity here is not only spatial but relational: it generates encounters, supports mutual care and creates the conditions for community-based forms of wellbeing (Klinenberg, 2018; Sennett, 2012). Through these mechanisms, Cultural Welfare symbolically and concretely counters the “broken windows” effect, fostering collective responsibility and neighborly ties. The beneficiaries are residents of all ages, with particular attention to children, older adults living alone and other vulnerable groups, for whom access to cared-for and animated spaces has central value.

The interviews show how, in organizations' own accounts, the constant presence of cultural and associative actors in an urban area perceived as unsafe has contributed to transforming how that place is viewed, with organizations reporting a reduction in situations of decay, problematic behaviors and abandonment. Regeneration here does not occur only through structural interventions; it also takes shape through a sustained on-site cultural presence. As a space becomes cleaner, more frequented and more inhabited, the sense of safety increases accordingly:

*Residents in the district have told us that they are no longer afraid to go out or return home at certain hours. This used to be a dark street, with problematic nighttime activity. Since we are open until about one or one-30 in the morning, those presences are no longer visible. We treat this area as if it were our home, and we respond immediately whenever there are reports of neglect or dirt. The street has improved considerably, not only in relation to problematic behaviors but also in terms of cleanliness. When a street is deserted, people tend to use it in problematic ways. For instance, at the end of the street, until 2 years ago, it was common to see people using the area to take drugs. Today this happens maybe once a month. A steady presence in the area discourages its use as a space of decay*. (INT04; translated from Italian by the authors with the assistance of artificial intelligence)

Another interview confirms that cultural events, exhibitions, street festivals and convivial moments can generate encounters among different generations and social groups. In this case, urban regeneration is not only material but profoundly relational and symbolic. Public space becomes a site of cohesion and belonging, where boundaries between social worlds that normally remain separate among young and older residents, long-term inhabitants and newcomers such as students or migrants, committees with differing visions are temporarily suspended in favor of shared participation and exchange. This creates fertile ground for dialogue, trust and the emergence of new networks and collaborations. The representative of one association engaged in the stewardship of a central green area emphasized, however, that the space cannot be described as a Cultural Welfare initiative in the narrow sense, showing that cultural practices, while central, are embedded in a broader ecology of tools, relationships and social policies:

*The first goal remains building relationships. It is a place where sociality is cultivated, and sociality is wellbeing. We did not only use cultural tools, we used many different tools. The garden evolved through intervention perspectives that cannot be rigidly contained within a strict model of Cultural Welfare, because we adapted our modes, means, and initiatives to each situation. There was a period of civic assistants, a period of training for parents, a period dedicated to bicycles, where we taught children to repair them. I would define it as a place aimed at coexistence and social safety through a variety of social policies. The cultural component has been decisive and important, but not the only one*. (INT02; translated from Italian by the authors with the assistance of artificial intelligence)

This reflection should not be read as contradicting the Cultural Welfare framework. Rather, it demonstrates its very innovative potential: culture is not an isolated domain but one that interacts with other dimensions of community life: education, care, social support, civic engagement. The case shows that cultural participation operates alongside other practices to generate proximity, trust and shared responsibility, while retaining the intrinsic value of the arts (Paltrinieri and Allegrini, 2020). This is precisely the relational co-constitution of the “urban,” the “social” and “innovation” that (McFarlane et al. 2023) describe: the innovation is not the cultural output as such, but the way culture is brought into relation with the other registers of neighborhood life.

A further dimension of this relational mode of regeneration concerns how informal networks of proximity reconfigure the everyday use of public space, particularly for children and adolescents. In another interview (INT09), a residents' committee member describes practices of collective supervision of children's independent mobility, walking to school, running small errands, reaching a friend's house, not by removing risk through (digital) surveillance, but by distributing watchful attention across a dense web of neighbors, shopkeepers and older residents who recognize and informally look out for local children. This form of diffuse, low-key supervision allows children to experience autonomy at an age when, in many other urban contexts, such independence would be considered dangerous. Importantly, interviewees frame this not as a spontaneous given, but as something that has to be actively cultivated and handed down: initial phases of collective accompaniment, such as adults cycling routes to school together with children at the start of the school year, are described as necessary scaffolding before unsupervised mobility becomes possible. The relational fabric of the neighborhood thus operates as a substitute for technological or infrastructural security measures, producing a felt sense of safety that several residents explicitly contrast with the experience of areas where this social infrastructure is thinner.

This preference for relational over technological solutions to perceived insecurity also surfaces explicitly in residents' encounters with municipal proposals for urban safety. In one notable instance, a residents' committee was offered the installation of surveillance cameras in a square experiencing low-level disorder, and instead requested a program of cultural animation and continuous presence in the square, carried out in partnership with a local theater and an intercultural association. From the committee's perspective, “cameras would have further isolated the space rather than making it more lively or trustworthy; what the square needed was not monitoring but inhabitation” (INT09; translated from Italian by the authors with the assistance of artificial intelligence). This episode illustrates the argument developed throughout this section: urban regeneration through Cultural Welfare reframes security as an emergent property of social presence and cultural activity rather than as a good to be supplied through digital technologies or control. As a single account, however, it cannot by itself establish a general relationship between cultural presence and urban safety. It also resonates with the relational understanding of urban social innovation outlined earlier (McFarlane et al., 2023): the same municipal square is reterritorialized by residents as a site of encounter rather than of risk, and the “urban” here is actively composed through this contestation between control-oriented and relational understandings of safety.

These dynamics, however, are not evenly distributed across the city. Interviews conducted in different areas of the same administrative district reveal markedly different intensities of relational regeneration, even where formal opportunities for participation, calls for proposals, co-design tables, collaboration agreements are nominally similar. In some neighborhood contexts, organizations describe a self-sustaining cycle in which cultural initiatives attract volunteers, generate intergenerational exchange and progressively densify the relational fabric of the area. In others, structurally comparable spaces struggle to overcome an aging membership base, a thin and largely passive constituency, and a perceived disconnection between residents and the very space meant to serve them. This unevenness should not be read as a simple matter of organizational effort or institutional support; rather, it reflects the situated and historically sedimented ways in which the urban is variously composed, inhabited and made meaningful by different communities (McFarlane et al., 2023), as well as the role of uncertainty and pre-existing social capital in enabling or constraining socially creative milieus (von Schnurbein et al., 2023). The same city, and even the same district, can therefore contain both vibrant and comparatively dormant configurations of Cultural Welfare, underscoring the limits of treating “the urban” as a homogeneous scale of analysis.

The findings reveal a consistent pattern across the two domains analyzed. Whether Cultural Welfare works to reduce inequalities and widen participation, or to regenerate the shared spaces of the city, its distinctive contribution lies in the same underlying operation: the production of shared meanings through artistic and cultural practices, and the relational fabric these meanings sustain. Inclusion and regeneration are thus two faces of one process, since participation is continuously produced through places, and places become liveable through the relations that cultural practices activate. What qualifies Cultural Welfare as a form of urban social innovation is precisely this capacity of culture to generate shared meanings that enable an alternative imagination through which residents can collectively reshape and inhabit the city. The following section discusses this argument theoretically, reading the Bologna case through the conceptual structure of urban social innovation in order to specify what kind of innovation Cultural Welfare represents and what orientation of the “social” it enacts.

## 5 Discussion

The analysis reconstructs the Cultural Welfare ecosystem of Bologna as a form of city-scale urban social innovation. Rather than treating Cultural Welfare as an “add-on” to existing welfare provision, interviewees describe it as a situated ecosystem of cultural practices that reconfigures how support, participation and everyday urban liveability are produced. Within this perspective, Cultural Welfare operates through two interconnected urban functions. First, it works as an inclusion-oriented infrastructure that reduces inequalities by expanding access to cultural participation, enabling voice and agency for groups exposed to marginalization, and strengthening community-based organization. Second, it acts as a cohesion-oriented infrastructure that contributes to the everyday maintenance and regeneration of public space, countering forms of urban decay through presence, care and relational mediation. These two functions are analytically distinct but empirically intertwined. What connects them is a shared relational and symbolic ground: both work by producing recognition, participation and shared meaning through cultural practices. Inclusion expands the capacity to participate, to be recognized and to act; regeneration reshapes the collective conditions under which urban space can be inhabited and collective life reimagined.

Read through the conceptual structure of urban social innovation, the analysis of data allows us to close the gap between the normative principles of urban social innovation and a situated empirical case. The three principles identified by Moulaert et al. (2013) are all legible in the data, but in a distinctively cultural register. The first principle, meeting a genuine need, appears in the way organizations detect needs that are not reducible to material deprivation: symbolic recognition, relational isolation, gendered discrimination, intergenerational fragmentation. The second, generating new forms of social and institutional relations, appears in the bottom-linked arenas that connect associations, schools, anti-violence centers, social services and residents' committees around shared cultural practices. The third, collectively empowering people, especially the marginalized, appears as what we have termed cultural capacitation and the production of collective cultural capital (Paltrinieri, 2025): the expansion of capabilities (Sen, 1999) and recognition (Fraser, 2000) through which participants reinterpret their position and inhabit urban space with greater voice and agency. Cultural capacitation, in other words, is the culturally mediated form that the empowerment principle takes in the case of Bologna.

Yet the contribution of this article is not simply to confirm Moulaert's principles in a new setting. Following McFarlane et al. (2023), we read Cultural Welfare as a relational co-constitution of the “urban,” the “social” and “innovation” that differs across the city. Two findings make this relational reading indispensable. The first is the explicit refusal, by an association, to be classified as Cultural Welfare “in the narrow sense” (INT02). Far from undermining our argument, this refusal substantiates it: the innovation does not reside in cultural activity as a discrete sector, but in the way culture is brought into relation with education, care, civic engagement, participation and the everyday governance of space. The second is a residents' committee's rejection of surveillance cameras in favor of activities carried out with a theater group and continuous artistic presence. Here the square is composed differently: rather than being monitored through digital surveillance, it is filled with cultural activity, and it is precisely this that allows residents to reterritorialize it as a place of encounter and shared life rather than a site of risk (INT09). What makes the square safe is not surveillance but the shared meanings and encounters that cultural practices generate within it. This is urban social innovation as the “drilling down” that McFarlane et al. advocate: the urban is practiced rather than given, through cultural participation in the case of Bologna, and its transformative potential lies in the shared and symbolic work of sustaining relations and meanings rather than in the production of novel outputs imposed by neoliberal logic. Yet this episode also invites a more cautious reading. Choosing cultural participation over surveillance cameras resolves the immediate dispute over the square, but it does not settle the underlying political question of how policing, security and public resources should be allocated across the city; in this sense, the episode may equally be read as one in which a properly political conflict, over who controls public space and through what means, is channeled into a register of cultural mediation that leaves the broader distribution of municipal security resources unchanged. We do not think this reading cancels the first: the residents' committee's choice was an exercise of agency over how their own square would be inhabited. But it is worth being explicit that Cultural Welfare's capacity to reframe conflicts in relational terms is not the same as its capacity to resolve the political. On this basis, our central theoretical claim is that Cultural Welfare constitutes a relational form of urban social innovation whose specific orientation of the social is the regeneration, through cultural means, of the relational fabric eroded by contemporary individualization. What is distinctive is not only what is regenerated, proximity, trust, mutual recognition, but how: it is through the production of shared meanings, symbolic recognition and collective imagination that cultural practices rebuild social bonds among people increasingly individualized and disembedded from collective life (Bauman, 2000; Beck and Beck-Gernsheim, 2002). This rebuilding is culturally mediated: it is the symbolic and expressive force of art and culture that generates the encounters, meanings and forms of recognition on which relational proximity depends. The value Cultural Welfare generates is precisely this culturally produced relational infrastructure, which countervails the abstraction of late-modern urban existence and which Klinenberg (2018) identifies as the often invisible substrate of liveable cities. In this sense, the article extends urban social innovation theory toward a dimension the field has tended to under-theorize: culture functions here not as content or output, but as an infrastructure for relationality, the very medium through which the social is regenerated.

This reframing also speaks to debates on urban transition. Rather than approaching transition exclusively through environmental or infrastructural lenses, the Bologna case foregrounds its socio-relational dimension: a city's capacity to become and remain liveable over time depends not only on environmental performance or economic dynamism, but on the strength of its relational infrastructures and on how widely opportunities for participation and recognition are shared. As anticipated in the Introduction, it is important, here, to distinguish the argument of this article from the influential body of work on the “creative class” and “culture-led regeneration” Florida, (2002; Miles and Paddison, 2005). That literature concerns culture as a driver of economic growth, the attraction of creative and knowledge workers, place marketing and property-led redevelopment, and has been extensively criticized for reproducing, rather than mitigating, socio-spatial inequality and gentrification (Peck, 2005). Community-based Cultural Welfare belongs to a different paradigm altogether. It is not concerned with culture as an engine of competitiveness or with a “creative class” of cultural professionals, but with cultural participation as an infrastructure of proximity, care and recognition, produced from below by and for heterogeneous communities. Even (Florida 2017), revising his earlier thesis, acknowledges that the clustering of talent and economic activity that powers urban growth also deepens inequality and segregation; yet the remedies he envisages remain largely those of economic and urban policy, reforming zoning and taxation, investing in density, expanding affordable housing. Cultural Welfare operates on a different and complementary terrain, one that such top-down instruments do not reach, yet its effects are no less material. Working through everyday cultural practice, it reclaims degraded and abandoned spaces, restores the safe and shared use of streets and squares, curbs the low-level disorder that fiscal and planning measures rarely touch, and sustains the dense webs of mutual attention on which residents' daily security depends. What is produced is a concrete relational infrastructure: physical spaces returned to collective use, social ties that make neighborhoods navigable and inhabitable, and forms of recognition that let people occupy urban space with voice and agency. In this sense, Cultural Welfare is one of the everyday, bottom-up mechanisms through which the material and relational conditions of urban liveability are jointly sustained.

These material effects, however, operate at a specific scale, and it is important not to overstate their reach or to assume their continuity. The organizations we studied sustain relational infrastructures under conditions of considerable fragility. Much of their work depends on project-based funding secured through competitive calls, which makes activities vulnerable to interruption once a grant cycle ends and rarely guarantees the continuity on which relational ties depend. This dependence, moreover, is not evenly distributed: interviewees describe a marked asymmetry in the capacity to access public and private calls, as larger associations, with dedicated administrative staff, accountants and technical offices, are better positioned to navigate the bureaucratic and technical requirements of funding calls, such as impact studies, safety plans and financial reporting, while smaller and more informal organizations, run entirely by unpaid volunteers, often lack the administrative capacity to compete and, in some cases, abandon planned initiatives altogether rather than absorb these costs. As one interviewee put it, those who win the funding “are the large associations, because they have the means, plenty of resources, cooperatives with strong backing” (INT01; translated from Italian by the authors with the assistance of artificial intelligence); another described the experience of chronic underfunding directly, requesting a given amount and receiving only a fraction of it, while still being expected to deliver the originally proposed activity in full (INT12). Cultural Welfare's relational infrastructure, in other words, is not distributed neutrally across the third sector: it tends to consolidate around organizations that are already better resourced and more experienced in navigating institutional procedures, a dynamic that risks reproducing, rather than mitigating, inequalities among third-sector actors themselves. Much of the administrative labor this requires, reporting, compliance, technical documentation, falls on unpaid volunteers, compounding a second fragility: the volunteer and associative base on which these organizations rely is itself thinning, as several interviewees describe an aging membership and the difficulty of involving younger residents, increasingly absorbed by work and time pressures. These are not incidental difficulties but structural conditions of third-sector cultural action, and they mean that Cultural Welfare mitigates, without resolving, the wider pressures, economic restructuring, uneven governance capacity, and the deepening urban inequalities of contemporary cities (Florida, 2017), within which it operates, while also raising the question of how much of the responsibility for social cohesion this arrangement quietly transfers from public administration to unremunerated civil society labor. Its contribution is therefore not an alternative to structural policy on housing, inequality and long-term governance, from which medium-sized cities such as Bologna are not exempt, but a complement to it: sustaining, at the everyday scale, the relational and spatial conditions that allow a city to remain liveable and inclusive over time, an often under-recognized dimension of urban sustainability. This caution about scale is reinforced by a further finding: the marked unevenness of relational regeneration, even within a single administrative district, cautions against any assumption that Cultural Welfare can be replicated on command. This resonates with von Schnurbein et al.'s (2023) account of socially creative milieus, in which dense networks of relations and trust, themselves both a condition and a product of collaborative practice, allow social innovation to emerge and to absorb uncertainty as a productive resource rather than a threat. Where cultural initiatives find such relational density, they reinforce it in a self-sustaining cycle; where the relational fabric is thinner, structurally comparable initiatives struggle to take root. This does not imply that a fixed stock of social capital must pre-exist for Cultural Welfare to work, our analysis has shown, on the contrary, that cultural practices actively generate relational ties; but rather that the pace and depth of this generative process are shaped by the situated, historically sedimented conditions of each area, and cannot be engineered uniformly through formal participatory instruments alone.

Beyond questions of scale, a further limitation concerns decision-making power itself. Interviewees describe co-design and co-programming with the municipality as operationally substantive: information flows, calls for proposals and opportunities for consultation are, by most accounts, reasonably accessible. Yet our data speak more directly to this operational dimension than to the allocation of resources and priorities at the policy level, where final decisions largely remain with the municipal administration. We can therefore describe organizations' participation in shaping how Cultural Welfare is implemented, but not confirm, on the basis of our data, that this participation extends to a redistribution of decision-making authority over funding priorities or urban policy more broadly.

## 6 Conclusion

This article has examined how third-sector and cultural actors across the medium-sized city of Bologna engage with intersecting processes of urban transformation, and has argued that their work can be understood as a relational form of urban social innovation. Drawing on 26 interviews with organizations operating across the city, we have shown that Cultural Welfare unfolds along two interconnected functions: reducing inequalities and promoting inclusion, and fostering culture-led regeneration for social cohesion. Both are best understood not as service provision but as the production and maintenance of a relational infrastructure through artistic and cultural practices. What distinguishes this form of social innovation is precisely its cultural register: it is by generating shared meanings, symbolic recognition and collective imagination that cultural practices rebuild the proximity, trust and mutual recognition eroded by contemporary individualization and social isolation. Culture, in this sense, functions not as content or output, but as the very medium through which the social fabric of the city is regenerated.

The article makes three contributions. Empirically, it extends the study of urban transition in medium-sized European cities beyond environmental and infrastructural framings, foregrounding the socio-relational dimension of urban change and the often invisible labor that cultural actors perform to hold the urban fabric in relation. Conceptually, it brings the Cultural Welfare paradigm into systematic dialogue with urban social innovation scholarship, showing that the cultural capacitation and collective cultural capital at the heart of Cultural Welfare are the culturally mediated form taken by the empowerment principle of urban social innovation. Theoretically, and most importantly, it argues that Cultural Welfare constitutes a relational form of urban social innovation whose specific orientation of the social is the regeneration of the relational fabric eroded by contemporary individualization and isolation. In foregrounding culture as an infrastructure for relationality, rather than as content, output or sectoral service, the article extends urban social innovation theory toward a cultural and relational dimension that its dominant, entrepreneurially oriented strands have left under-theorized, strands that define social innovation primarily through cross-sector value creation and the diffusion of scalable solutions (Phills et al., 2008), rather than through the situated cultural work of sustaining relations.

However significant these contributions, they are subject to clear limits. The study is based on a single, paradigmatic case and aims at analytical rather than statistical generalization; the marked intra-urban unevenness we document suggests that the conditions enabling Cultural Welfare cannot be assumed to travel unchanged to other settings. Moreover, Cultural Welfare mitigates but does not resolve the structural pressures such as housing, inequality, and governance capacity that reshape contemporary cities. Future research could pursue comparative work across medium-sized cities to distinguish place-specific from structural factors, and examine longitudinally whether and under what conditions these relational infrastructures consolidate or erode over time. What the Bologna case makes visible, in all its uneven diversity, is that the liveability of a city is renegotiated not only through plans and markets, but through a situated cultural work of holding a community in relation.

These contributions, however, must be read alongside a number of limitations. A related gap concerns hybrid and digital dimensions of participation. Although our fieldwork covers 2024–2026, our interview guide did not systematically probe how communication platforms, messaging applications, social media and digital organizational practices shape recruitment, continuity, visibility or exclusion; where interviewees mentioned these tools, it was incidental to other themes rather than the focus of sustained inquiry, and we do not have systematic evidence on this dimension of participation. A further limitation concerns whose voices this study is able to represent, and what kind of evidence can support the claims examined throughout this article. Our empirical material derives entirely from interviews with organizational representatives, activators and facilitators, and does not include the perspectives of the residents and participants who take part in these activities, still less those of residents who do not; nor did we triangulate these accounts with observational fieldnotes, municipal or organizational records, attendance and participation data, neighborhood-level indicators, project evaluations, or evidence of continuity beyond specific funding cycles. This is a substantial epistemic limitation: people experiencing marginalization or isolation appear in our account largely through the interpretive categories of the organizations that work with them, and claims about safety, cohesion or reduced inequality should be read as organizationally reported and perceived outcomes rather than externally verified ones. Interviewing the full range of participants and non-participants across 26 organizations was beyond the scope of a study of this design; addressing this gap would require a differently structured research design, most plausibly organized around a smaller number of sites studied in greater depth.

Relatedly, the qualitative and largely retrospective character of our interviews limits our ability to assess long-term outcomes or to establish continuity once specific funding streams end. Because Cultural Welfare initiatives are also more likely to take root where relational density and social capital are already comparatively strong, our design cannot fully disentangle the effects generated by cultural practices themselves from the pre-existing relational conditions in which those practices are embedded.

## Data Availability

The original contributions presented in the study are included in the article/supplementary material, further inquiries can be directed to the corresponding author.
